# Impact of Periodontal Disease on the Quality of Life of Diabetics Based on Different Clinical Diagnostic Criteria

**DOI:** 10.1155/2012/986412

**Published:** 2012-09-29

**Authors:** Aline Mendes Silva de Pinho, Carolina Marques Borges, Mauro Henrique Nogueira Guimarães de Abreu, Efigênia Ferreira e Ferreira, Andréa Maria Duarte Vargas

**Affiliations:** ^1^Department of Social and Preventive Dentistry, School of Dentistry, Federal University of Minas Gerais, 32170-901 Belo Horizonte, MG, Brazil; ^2^Rua Maria Heilbuth Surette, 528 /102-Buritis, 30575-100 Belo Horizonte, MG, Brazil

## Abstract

The aim of this study was to determine the impact of periodontal disease on the quality of life of individuals with diabetes according to different clinical criteria (I-AAP, II-Beck, III-Machtei, IV-Lopez, V-Albandar, VI-Tonetti, and VII-CPI). This cross-sectional study sampled 300 individuals in Belo Horizonte, Brazil. The Oral Health Impact Profile was used to measure the impact of periodontal disease on quality of life. Prevalence of periodontal disease was 35.3%, 30.7%, 35.0%, 9.7%, 92.3%, 25.3%, and 75.3% using criteria I, II, III, IV, V, VI, and VII, respectively. The III-Machtei (*P* = 0.043) and IV-Lopez (*P* < 0.001) criteria were associated with OHIP-14; functional limitation was associated with IV-Lopez (*P* = 0.006) and V-Albandar (*P* = 0.018) criteria. Pain was only associated with V-Albandar criteria (*P* < 0.001). Psychological discomfort was associated with the IV-Lopez (*P* = 0.018) criteria. Physical disability was associated with the IV-Lopez (*P* = 0.047) and V-Tonetti (*P* = 0.046) criteria. Being handicapped was associated with the I-AAP (*P* = 0.025) and II-Beck (*P* = 0.041) criteria. Concepts of health and disease determined by clinical diagnostic criteria may influence the assessment of the impact of periodontal disease on diabetics' quality of life.

## 1. Introduction

Diabetes mellitus is one of the primary public health problems. Its chronic nature and the limitations it imposes contribute significantly to the increase in hospitalization, disability, and mortality rates. The occurrence of diabetes in population groups is linked mainly to socioeconomic factors, cultural factors, stress, and family predisposition [1]. In the year 2000, the prevalence of diabetes in the world was 171 million, and it is estimated that this number will reach 366 million by 2030 [2]. In Brazil, there are approximately 10 million people with diabetes.

Studies suggest that diabetes is a risk factor for periodontal disease, pointing out that the prevalence, incidence, and severity of periodontal disease are higher among individuals with diabetes in comparison to healthy individuals [10, 11]. The mechanisms by which diabetes influences periodontal disease include vascular abnormalities, neutrophil dysfunction, abnormalities in collagen synthesis, and genetic predisposition [12].

The most commonly employed clinical parameters for the diagnosis and classification of periodontal disease are measurements of the depth of periodontal pockets, clinical insertion loss, and bleeding upon probing, which often generate different information regarding the prevalence of the disease. There is no consensus in the scientific literature regarding a gold standard clinical diagnostic criterion for periodontal disease, which limits comparison among different studies [13]. 

The issue of accuracy of periodontal protocols of the National Health and Nutrition Examination Survey (NHANES) was discussed. The authors verified that the periodontal disease case used could lead to an underestimate of the prevalence of periodontal disease by 50% or more [14]. 

The change in the medical paradigm from focus on the presence or absence of disease to a broader view that considers the subjective experience of individuals regarding physical, social, and psychological wellbeing has allowed more detailed knowledge of the living conditions of the population [15]. Beside the need for the convergence of *clinical diagnostic criteria*, the concomitant use of nonnormative measures is important to determining whether periodontal disease causes an impact on people's daily lives. 

One of the first efforts to document this issue in Brazil was made by Leäo et al., who used the Dental Impact on Daily Living Index to measure periodontal health impacts on daily living.We made the highlighted change according to the list of references. Please check Roughly 60% of interviewed individuals reported some dissatisfaction with their quality of life [16]. It has been observed that studies relating the impact of periodontal disease on quality of life of diabetics are very scarce. Among these few studies, Drumond-Santana et al. evaluated the potential impact of periodontal disease on quality of life in diabetics. They found that *diabetics* with mild-to-moderate and advanced periodontal disease suffered a more negative impact on quality of life than those who were periodontally healthy or had gingivitis [17]. The Oral Health Impact Profile (OHIP) and its shortened version, the OHIP-14, are indexes that can be used for this purpose [18, 19].

Therefore, based on the hypothesis that the impact of periodontal disease on quality of life may vary depending on the clinical *diagnostic* criteria used for its diagnosis, the aim of this study was to test associations between different *clinical diagnostic criteria* for the determination of periodontal disease and its impact on the daily life of individuals with diabetes.

## 2. Methods

A cross-sectional study was carried out in 2005 in the city of Belo Horizonte, state capital of Minas Gerais, Brazil. The city has 2,412,937 inhabitants and occupies a strategic position on the geopolitical maps of Brazil and Latin America [20]. The study was approved by the Ethics Committee of the Federal University of Minas Gerais (Protocol no. 012/2004).

Sample calculation considered both type I and II errors. For this, we assumed a 95% confidence interval, 80% power of test, and parameters of diabetes and quality of life from Drumond-Santana et al. [17]. The sample calculation by comparison of proportions presented a higher number than those found by comparison of means. Thus, we conducted a different sample size calculation for each dimension of OHIP-14. This procedure assured, for a sample size of 300 individuals, a type I error = 0.05 and a power of test = 80% for all dimensions of OHIP-14, except for psychological disability, social disability, and handicap. 

We sampled 300 men and women over 30 years of age with a diagnosis of either type (I and II) of diabetes, and who were registered and assisted by the Belo Horizonte health care system. Diagnosis of diabetes was confirmed by municipality primary care physicians. In all participants the disease was under control. Sampling procedures were published elsewhere [21]. The city of Belo Horizonte has nine administrative districts with differences in the number of diabetic patients registered. Simple random sampling was performed for each district. Thus, the number of randomly selected patients was proportional to the number of diabetic individuals registered in each district. The number of individuals examined was determined by a sample calculation using a formula recommended by the World Health Organization [22] for the anticipated population proportion with the occurrence of disease. 

The inclusion criteria were individuals who had diabetes diagnose (either type I or II), up to 30 years old, were registered in the Belo Horizonte public health care system, were *dentate*, and had signed the informed consent. Additionally, teeth with gingival morphology damage, ill-adapted dental restorations, large cavitation, or third molars were excluded. 

### 2.1. Definition of Variables

The outcome was the impact of oral health on the quality of life of adults with diabetes measured by the modified Oral Health Impact Profile (OHIP-14), which has been validated for use in Brazil [23]. As the aim was to investigate the impact of periodontal disease, the word “gums” was added to 13 questions on the OHIP-14. This issue was adopted in accordance with Drumond-Santana et al. [17]. Responses were dichotomized as “no impact”, which only considered the option “never”, and “impact”, which considered the options “sometimes” and “always”. The administration of the OHIP-14 was carried out at basic health units of the public healthcare system. Periodontal disease was assessed by the following clinical criteria: gingival bleeding upon probing, probing depth, and clinical insertion loss. For the determination of gingival bleeding, a probe was introduced into the gingival pocket until the limit of its base, waiting 30 to 60 seconds for the determination of bleeding. Bleeding was recorded (presence or absence) using the modified Ainamo and Bay index [24]. Probing depth was determined from the measurement of the distance from the *gingival margin* to the bottom of the gingival *sulcus* or periodontal pocket. The values for each of the *surfaces* (mesial, distal, vestibular, and lingual) of all the teeth present were recorded. *Clinical attachment* loss was determined from the distance between the enamel-cementum junction and the bottom of the gingival *sulcus* or periodontal pocket. *Attachment* loss was measured using the enamel-cementum junction as the base, when visible.

### 2.2. Clinical Diagnostic Criteria for the Definition of Periodontal Disease

Seven sets of clinical criteria were used to diagnose periodontal disease and are described in Table 1. All exams were performed by one researcher (A.M.S.), who was submitted to the Kappa test (*κ*) for the determination of reliability (intraexaminer agreement). For the Kappa test, all clinical exams were performed at the College of Dentistry, Federal University of Minas Gerais. A total of 588 dental surfaces were clinically examined for the Kappa test (*κ*) calculation, with a one-week interval between each participant's first and second examinations. Results were satisfactory for all the clinical criteria: bleeding upon probing (0.79), probing depth (0.81), and clinical insertion loss (0.91). 

During the main study, clinical exams were performed in the dental offices of Brazilian National Health Care units under artificial light, using Williams periodontal probes (Hu-Friedy), mouth mirrors, and gauze. All norms of biosafety were followed. To obtain a CPI score, data collected were transformed according to CPI criteria. 

### 2.3. Data Analysis

The data were organized in a database using the Statistical Package for Social Sciences (SPSS) version 15.0 for Windows. Processing involved data codification, entry and editing. Absolute and relative frequencies were determined for all variables. Univariate analysis was carried out to determine the association of each set of clinical criteria used to estimate periodontal disease and the OHIP-14, using the Mann-Whitney, Kruskall Wallis and Pearson chi-square tests, with the significance level of *P* < 0.05.

## 3. Results

Of all study participants, the majority had diabetes type II (71.3%), were female (60%), age 55 or older (50.7%), living without a partner (57.3%), and had low educational level (71.3%) (Table 2).

**Figure 1:**
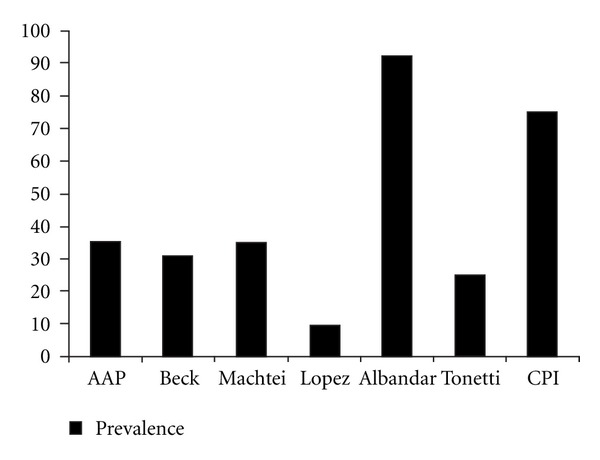
Prevalence of periodontal disease according to clinical diagnostic criteria. Belo Horizonte, 2005.

Table 3 presents the association between OHIP-14 overall results and periodontal disease, according to each one of the clinical diagnostic criteria. Only III-Machtei (*P* = 0.043) and IV-Lopez (*P* < 0.001) were statistically associated with *overall* OHIP-14 (Table 3). The prevalence of periodontal disease varied depending on the clinical criteria employed: 35.3% (I-AAP), 30.7% (II-Beck), 35.0% (III-Machtei), 9.7% (IV-Lopez); 92.3% (V-Albandar), 25.3% (VI-Tonetti), and 75.3% (VII-CPI) (Figure 1).

Regarding OHIP-14 subscales, functional limitation was associated with IV-Lopez (14.9% of diabetics with periodontal disease suffered an impact on quality of life; *P* = 0.006) and V-Albandar (28.4%, 8.2%, and 53.7% of diabetics with mild, moderate, severe periodontal disease, resp. suffered an impact on quality of life; *P* = 0.018); Pain was only associated with the V-Albandar criteria (28.1%, 14.5%, 49.2% of diabetics with mild, moderate, and severe periodontal disease, resp. suffered an impact on quality of life; *P* < 0.001). Psychological discomfort was associated with IV-Lopez (12.3% of diabetics with periodontal disease suffered an impact on quality of life; *P* = 0.018). Physical disability was associated with IV-Lopez (13.2% of diabetics with periodontal disease suffered an impact on quality of life; *P* = 0.047) and V-Tonetti (30.6% of diabetics with periodontal disease suffered an impact on quality of life; *P* = 0.046) criteria. Social disability was only associated with IV-Lopez (13.0% of diabetics with periodontal disease suffered an impact on quality of life; *P* = 0.056). A handicap was associated with I-AAP (47.0% of diabetics with periodontal disease suffered an impact on quality of life; *P* = 0.025) and II-Beck (40.9% of diabetics with periodontal disease suffered an impact on quality of life; *P* = 0.041) criteria (Tables 4 and 5).

## 4. Discussion

The choice of one of the different sets of clinical criteria for the diagnosis of periodontal disease is not a simple task, since there is no consensual recommendation regarding the use of these measures [3–5]. The measurement of health or disease does not go beyond the tooth or site and controversial measures impede the determination of an individual as either healthy or sick. For instance, in our study the prevalence of periodontal disease ranged from 9.7% (IV-Lopez) to 92.3% (V-Albandar), depending on which set of clinical diagnostic criteria was used. 

This disparity calls for reflection. Some of the clinical diagnostic criteria used in the present study consider the result of the exam by the number of affected sites, whereas others consider the number of affected teeth or a combination of teeth and sites. Moreover, depending on the manner in which the measurement is determined, a single tooth may have four or six sites. Manau et al. investigated whether the application of different definition criteria of periodontal disease had an influence on the significance of the association between periodontal disease and prematurity or low birth weight. They used fourteen definitions of periodontal disease and results showed that significance of the association between periodontal disease and pregnancy outcomes may be determined by the periodontal disease definition or measurement used [13]. A similar study was carried out involving cases and controls in order to compare four definitions of periodontal disease and respective associations with premature birth/low birth weight; the authors concluded that the magnitude of the association varied depending on the definition of periodontal disease employed [25].

Two clinical criteria of periodontal disease (Machtei and Lopez) were statistically associated with the OHIP-14 overall score. The Machtei criteria may be considered the broadest for determining the prevalence of periodontal disease, as it considers insertion loss ≥ 6 mm in two or more sites and probing depth ≥ 5 mm in one or more sites for the diagnosis of the disease. The Lopez criteria appear to be more cautious regarding the extension of periodontal disease, as they consider four or more teeth with at least one site with probing depth ≥ 4 mm and insertion loss ≥ 3 mm. 

It is appropriate to highlight that Community Periodontal Index (CPI) was statistically associated with neither the overall OHIP-14 nor with any of its *subscales*. CPI is recommended as an epidemiological tool by the World Health Organization (WHO), and it has been widely used internationally. In our study, we were able to classify the worst CPI criteria code 3 or 4 (with pockets). If the patient had only one site probing ≥ 4 mm, he/she was diagnosed as having periodontal disease. This may explain why there was no association between impact on quality of life and periodontal disease. 

The test of the association of each of the seven sets of criteria and the OHIP-14 dimensions showed that the Lopez criteria were statistically associated with functional limitation, psychological discomfort, physical disability, and social disability (limitrophe *P* value for this last one). This evidence may be explained considering that the more strict a clinical criterion is, the higher the chances are of it having an association with negative impact on quality of life. The Albandar criterion was only associated with pain. Psychological disability was not associated with any of sets of clinical criteria for the diagnosis of periodontal disease. Depending on the clinical criteria used, there were differences in the impact of periodontal disease on quality of life, even when measured by the same questionnaire (OHIP-14).

Different cut-off points for probing depth and insertion loss imply differences in prevalence rates and the results of tests on associated factors. Criteria for the definition of the presence and absence of periodontal disease are controversial, and there is a clear need to establish a cut-off point between health and disease for periodontics, as well as establish the meaning of the disease for individuals. Towards this end, some studies have adopted more rigorous criteria to minimize overestimated rates of periodontal disease and its association with quality of life. Severe periodontal disease, measured by Beck criterion, will negatively impact on the diabetic's life, as measured by OHIP-14 [17]. 

It is worth noting that the majority of studies published so far comprise the issue of periodontal disease and quality of life in individuals without diabetes. Bernabé and Marcenes (2010) investigated the association between quality of life and periodontal disease among 3,122 British adults and found that they were inversely associated in spite of socioeconomic conditions, demographic factors, and clinical conditions [26]. Among the few studies which focused on the relation between diabetes and quality of life, Sandberg and Wikblad (2003) pointed out that having diabetes played an important role in the domains physical functioning, role functioning physical, general health and social functioning, when compared to patients without a history of diabetes [27].

Although our study included a representative sample of diabetic patients in the public healthcare system of Belo Horizonte, Brazil, it is important to point out some limitations of this research. First, our study is cross-sectional, which does not allow any inference with regard to causality. Second, our sample size does not give us reasonable values for type I and II errors for the dimensions “psychological disability,” “social disability” and “handicap.” The main issue raised by the results of the present study is that the definition of a disease established by a given set of clinical diagnostic criteria may either overestimate or underestimate the chances of an individual being considered sick or healthy. Likewise, the impact periodontal disease has on quality of life may vary depending on the definition of the disease established by one of the seven sets of diagnostic criteria analyzed here. Additionally, we did not register the mean duration of diabetes, and therefore were not able to consider development of comorbidities that negatively impact quality of life.

As health and sickness are terms that correspond to unique experiences laden with subjectivity, it seems unlikely that these terms are recognized in the totality of their meanings [28]. The concrete phenomenon of becoming ill, self-perception of the individual, and professional diagnosis most often fail to coexist in harmony [29]. This means that the same disease may be seen from different standpoints, the consequences of which are the differences identified in the impact that periodontal disease has on the daily life of affected individuals.

## 5. Conclusion

The concept of health or disease as determined by clinical diagnostic criteria may influence the results of the assessment of the impact of periodontal disease on quality of life of diabetics, while the perception of affected individuals is unique. Studies directed at the establishment of standardized criteria for the diagnosis of periodontal disease are needed. Such studies should be focused on the health-disease process, including its social determination. A reference standard for epidemiological studies addressing periodontal disease could contribute to the organization of health actions in the field of collective health.

## Figures and Tables

**Table 1:** Description of clinical diagnostic criteria for periodontal disease.

Clinical diagnostic criteria	Description
(I) AAP	Presence of disease when a person has, at least, one tooth probing depth ≥ 4 mm and clinical insertion loss ≥ 4 mm at the same site [3].
(II) Beck	Attachment loss ≥ 5 mm in four or more sites and at least one of these sites with probing depth ≥ 4 mm [4].
(III) Machtei	Attachment loss ≥ 6 mm in two or more teeth and probing depth ≥ 5 mm in one or more sites [5].
(IV) Lopez	Four or more teeth with at least one site with probing depth ≥ 4 mm and insertion loss ≥ 3 mm [6].
(V) Albandar	Classifies periodontal disease as mild (1 or more teeth with probing depth ≥ 3 mm), moderate (1 or more teeth with probing depth ≥ 5 mm or 2 or more teeth with probing depth ≥ 4 mm), and severe (2 or more teeth with probing depth ≥ 5 mm or 4 or more teeth with probing depth ≥ 4 mm) [7].
(VI) Tonetti	Considers attachment loss ≥ 3 mm in two or more nonadjacent teeth or insertion loss ≥ 5 mm in 30% in of teeth [8].
(VII) Community Periodontal Index (CPI)	Considers the worst condition encountered in six sites evaluated and used the following four codes: 0 = healthy; 1 = absence of pockets, bacterial plaque retention factors, or bleeding following probing; 2 = depth as much as 3 mm and presence of bacterial plaque retention factors; 3 = pockets with probing depth between 4 and 5 mm; 4 = probing depth ≥ 6 mm [9]

**Table 2:** Sample characterization. Belo Horizonte, 2005.

Variable	*N* (%)
Gender	
Male	120 (40)
Female	180 (60)
Age	
30–54 years old	148 (49.3)
≥55 years old	152 (50.7)
Marital status	
With partner	128 (42.7)
Without partner	172 (57.3)
Income	
≤R$ 400	150 (50)
>R$ 400	150 (50)
Educational background	
Bachelor/high School	48 (16)
Elementary/none	252 (84)
Type of diabetes	
Type I	86 (28.7)
Type II	214 (71.3)

**Table 3:** Mean, standard deviation, median, and *P* value, OHIP-14 among diabetics. Belo Horizonte, 2005.

Diagnostic criteria		Mean (± SD)	Median	*P* value
(I) AAP	Healthy	7.56 (5.86)	6.45	0.120
	Periodontal disease	8.79 (6.43)	7.72	

(II) Beck	Healthy	7.57 (5.86)	6.70	0.099
	Periodontal disease	8.94 (6.51)	7.62	

(III) Matchei	Healthy	7.49 (5.99)	6.40	0.043
	Periodontal disease	8.92 (6.18)	8.10	

(IV) Lopez	Healthy	7.57 (5.92)	6.50	<0.001
	Periodontal disease	11.91 (6.34)	12.38	

(V) Albandar	Healthy	7.05 (4.70)	5.72	0.265
	Mild PD	7.32 (6.22)	5.60	
	Moderate PD	7.65 (6.39)	7.00	
	Severe PD	8.64 (6.09)	8.07	

(VI) Tonetti	Healthy	7.66 (5.94)	6.81	0.130
	Periodontal disease	8.97 (6.45)	8.16	

(VII) CPI	Healthy	7.87 (5.52)	8.06	0.470
	Bleeding	7.16 (5.83)	4.98	
	Pocket 4-5 mm	7.73 (6.17)	6.90	
	Pocket ≥ 6 mm	8.69 (6.09)	7.06	

PD: Periodontal disease.

**Table 4:** Prevalence of periodontal disease among diabetics based on AAP, Beck, Matchei, Lopez, and Tonetti criteria and each of the seven OHIP subscales; *P* value; Belo Horizonte, 2005.

	OHIP subscales													
	Functional limitation		Pain		Psychological discomfort		Physical disability		Psychological disability		Social disability		Handicap	
Periodontal disease	No (%)	Yes (%)	No (%)	Yes (%)	No (%)	Yes (%)	No (%)	Yes (%)	No (%)	Yes (%)	No (%)	Yes (%)	No (%)	Yes (%)

AAP	*P* = 0.874		*P* = 0.880		*P* = 0.579		*P* = 0.787		*P* = 0.286		*P* = 0.191		*P* = 0.025*	
No impact	108 (65)	58 (34.9)	38 (65.5)	20 (34.5)	59 (67)	29 (33)	102 (65.4)	54 (34.6)	49 (70)	21 (30)	105 (68.2)	49 (31.8)	159 (67.9)	74 (32.1)
Impact	86 (64)	48 (35.8)	156 (64.5)	86 (35.5)	135 (63.7)	77 (36.3)	92 (63.9)	52 (36.1)	145 (63)	85 (37)	89 (61)	57 (39)	35 (53)	31 (47)

Beck	*P* = 0.631		*P* = 0.700		*P* = 0.411		*P* = 0.477		*P* = 0.465		*P* = 0.190		*P* = 0.041*	
No impact	117 (70.5)	49 (29.5)	39 (67.2)	19 (32.8)	64 (72.7)	24 (27.3)	111 (71.2)	45 (28.8)	51 (72.9)	19 (27.1)	112 (72.7)	42 (27.3)	169 (72.2)	65 (27.8)
Impact	91 (67.9)	43 (32.1)	169 (69.8)	73 (30.7)	144 (67.9)	68 (32.1)	97 (67.4)	47 (32.6)	157 (69.3)	73 (31.7)	96 (65.8)	50 (30.7)	39 (59.1)	27 (40.9)

Machtei	*P* = 0.214		*P* = 0.257		*P* = 0.312		*P* = 0.529		*P* = 0.317		*P* = 0.095		*P* = 0.397	
No impact	113 (68.1)	53 (31.9)	34 (58.6)	24 (41.4)	61 (69.3)	27 (30.7)	104 (66.7)	52 (33.3)	49 (70)	21 (30)	107 (69.5)	47 (30.5)	155 (66.2)	79 (33.8)
Impact	82 (61.2)	52 (38.8)	161 (66.5)	81 (33.5)	134 (63.2)	78 (36.8)	91 (63.2)	53 (36.8)	146 (63.5)	84 (36.5)	88 (60.3)	58 (39.7)	40 (60.6)	26 (39.4)

Lopez	*P* = 0.006*		*P* = 0.764		*P* = 0.018*		*P* = 0.047*		*P* = 0.082		*P* = 0.056		*P* = 0.858	
No impact	157 (94.6)	9 (5.4)	53 (91.4)	05 (8.6)	85 (96.6)	03 (3.4)	146 (93.6)	10 (6.4)	67 (95.7)	03 (4.3)	144 (93.5)	10 (6.5)	211 (90.2)	23 (9.8)
Impact	114 (85.1)	20 (14.9)	218 (90.1)	24 (9.9)	186 (87.7)	26 (12.3)	125 (86.8)	19 (13.2)	204 (88.7)	26 (11.3)	127 (87)	19 (13)	60 (90.9)	06 (9.1)

Tonetti	*P* = 0.583		*P* = 0.148		*P* = 0.932		*P* = 0.046*		*P* = 0.477		*P* = 0.424		*P* = 0.170	
No impact	126 (75.9)	40 (24.1)	39 (67.2)	19 (32.8)	66 (75)	22 (25)	124 (79.5)	32 (20.5)	50 (71.4)	20 (28.6)	118 (76.6)	36 (23.4)	179 (76.5)	55 (23.5)
Impact	98 (73.1)	36 (26.9)	185 (76.4)	57 (23.6)	158 (74.5)	54 (25.5)	100 (74.7)	44 (30.6)	174 (75.7)	56 (24.3)	106 (72.6)	40 (27.4)	45 (68.2)	21 (31.8)

*Statically significant.

**Table 5:** Prevalence of periodontal disease among diabetics based on Albandar and CPI criteria and each of the seven OHIP subscales; *P* value; Belo Horizonte, 2005.

	Albandar					Community periodontal index				
	Healthy (%)	Mild (%)	Moderate (%)	Severe (%)	*P* value	Healthy	Bleeding	Pocket = 4 or 5 mm	Pocket ≥ 6 mm	*P* value*
Functional limitation										
No impact	10 (6)	48 (28.9)	34 (20.5)	74 (44.6)	0.018*	14 (8.4)	28 (16.9)	69 (41.6)	55 (33.1)	0.506
Impact	13 (9.7)	38 (28.4)	11 (8.2)	72 (53.7)		15 (11.2)	21 (15.7)	46 (34.3)	52 (38.8)	

Pain										
No impact	03 (5.2)	18 (31)	10 (17.2)	27 (46.6)	<0.001*	06 (10.3)	10 (17.2)	25 (43.1)	17 (29.3)	0.726
Impact	20 (8.3)	68 (28.1)	35 (14.5)	119 (49.2)		23 (9.5)	39 (16.1)	90 (37.2)	90 (37.2)	

Psychological discomfort										
No impact	07 (8)	28 (31.8)	13 (14.8)	40 (45.5)	0.870	07 (8)	15 (17)	38 (43.2)	28 (31.8)	0.638
Impact	16 (7.5)	58 (27.4)	32 (15.1)	106 (50)		22 (10.4)	34 (16)	77 (36.3)	79 (37.3)	

Physical disability										
No impact	13 (8.3)	43 (27.6)	21 (13.5)	79 (50.6)	0.777	14 (9)	25 (16)	61 (39.1)	56 (35.9)	0.972
Impact	10 (6.9)	43 (29.9)	24 (16.7)	67 (46.5)		15 (10.4)	24 (16.7)	54 (37.5)	51 (35.4)	

Psychological disability										
Impact	05 (7.1)	24 (34.3)	10 (14.3)	31 (44.3)	0.699	08 (11.4)	11 (15.7)	30 (42.9)	21 (30)	0.654
No impact	18 (7.8)	62 (27)	35 (15.2)	115 (50)		21 (9.1)	38 (16.5)	85 (37)	86 (37.4)	

Social disability										
No impact	15 (9.7)	49 (31.8)	22 (14.3)	68 (44.2)	0.231	15 (9.7)	29 (18.8)	57 (37)	53 (34.4)	0.638
Impact	08 (5.5)	37 (25.3)	23 (15.8)	78 (53.4)		14 (9.6)	20 (13.7)	58 (39.7)	54 (37)	

Handicap										
No impact	18 (7.7)	71 (30.3)	34 (14.5)	111 (47.4)	0.675	25 (10.7)	40 (17.1)	89 (38.8)	80 (34.2)	0.534
Impact	05 (7.6)	15 (22.7)	11 (16.7)	35 (53)		04 (6.1)	09 (13.6)	26 (39.4)	27 (40.9)	

*Statiscally significant.

## References

[1] Brasil, Ministério da Saúde, Secretaria de Políticas de Saúde Plano de reorganização da atenção à hipertensão arterial e ao diabete mellitus. http://bvsms.saude.gov.br/bvs/publicacoes/miolo2002.pdf.

[2] World Health Organization http://www.who.int/en/.

[3] American Academy of Periodontology (2000). Parameter on chronic periodontal disease with advanced loss of periodontal support. *Journal of Periodontology*.

[4] Beck JD, Koch GG, Rozier RG, Tudor GE (1990). Prevalence and risk indicators for periodontal attachment loss in a population of older community-dwelling blacks and whites. *Journal of periodontology*.

[5] Machtei EE, Christersson LA, Grossi SG, Dunford R, Zambon JJ, Genco RJ (1992). Clinical criteria for the definition of ‘established periodontitis’. *Journal of Periodontology*.

[6] López NJ, Smith PC, Gutierrez J (2002). Higher risk of preterm birth and low birth weight in women with periodontal disease. *Journal of Dental Research*.

[7] Albandar JM (1990). A 6-year study on the pattern of periodontal disease progression. *Journal of Clinical Periodontology*.

[8] Tonetti MS, Claffey N (2005). Advances in the progression of periodontitis and proposal of definitions of a periodontitis case and disease progression for use in risk factor research: group C Consensus report of the 5th European workshop in periodontology. *Journal of Clinical Periodontology*.

[9] World Health Organization (1997). *Oral Health Surveys: Basic Methods*.

[10] Bjelland S, Bray P, Gupta N, Hirsch R (2002). Dentists, diabetes and periodontitis. *Australian Dental Journal*.

[11] Kasaj A, Zafiropoulos GG, Tekyatan H, Pistorius A, Willershausen B (2008). Periodontal disease status of pregnant women with diabetes mellitus. *Collegium Antropologicum*.

[12] Oliver RC, Tervonen T (1994). Diabetes—a risk factor for periodontitis in adults?. *Journal of Periodontology*.

[13] Manau C, Echeverria A, Agueda A, Guerrero A, Echeverria JJ (2008). Periodontal disease definition may determine the association between periodontitis and pregnancy outcomes. *Journal of Clinical Periodontology*.

[14] Eke PI, Thornton-Evans GO, Wei L, Borgnakke WS, Dye BA (2010). Accuracy of NHANES periodontal examination protocols. *Journal of Dental Research*.

[15] Leão AT, Sheiham A, Slade GD (1997). The dental impact on daily living. *Measuring Oral Quality of Life*.

[16] Leäo ATT, Cidade MC, Varela JR (1998). Impactos da saúde periodontal na vida diária. *Revista Brasileira de Odontologia*.

[17] Drumond-Santana T, Costa FO, Zenóbio EG, Soares RV, Santana TD (2007). Impact of periodontal disease on quality of life for dentate diabetics. *Cadernos de Saude Publica*.

[18] Slade GD, Spencer AJ (1994). Development and evaluation of the oral health impact profile. *Community Dental Health*.

[19] Slade GD (1997). Derivation and validation of a short-form oral health impact profile. *Community Dentistry and Oral Epidemiology*.

[20] Prefeitura Municipal de Belo Horizonte. http://portalpbh.pbh.gov.br/pbh/.

[21] Silva AM, Vargas AMD, Ferreira E, de Abreu MHNG (2010). Periodontitis in individuals with diabetes treated in the public health system of Belo Horizonte, Brazil. *Revista Brasileira de Epidemiologia*.

[22] Lwanga SK, Lemeshow S *Sample Size Determination in Health Studies: A Pratical Manual*.

[23] de Oliveira BH, Nadanovsky P (2005). Psychometric properties of the Brazilian version of the oral health impact profile—short form. *Community Dentistry and Oral Epidemiology*.

[24] Ainamo J, Bay I (1975). Problems and proposals for recording gingivitis and plaque. *International Dental Journal*.

[25] Gomes-Filho IS, Cruz SS, Rezende EJC (2007). Exposure measurement in the association between periodontal disease and prematurity/low birth weight. *Journal of Clinical Periodontology*.

[26] Bernabé E, Marcenes W (2010). Periodontal disease and quality of life in British adults. *Journal of Clinical Periodontology*.

[27] Sandberg GE, Wikblad KF (2003). Oral health and health-related quality of life in type 2 diabetic patients and non-diabetic controls. *Acta Odontologica Scandinavica*.

[28] Czeresnia D, Czeresnia D, Freitas CM (2008). O conceito de saúde e a diferença entre prevenção e promoção. *Promoção da saúde. Conceitos, reflexões, tendências*.

[29] Caponi S, Czeresnia D, Freitas CM (2008). A saúde como abertura ao risco. *Promoção da saúde. Conceitos, reflexões, tendências*.

